# Letter to the Editor: Randomized Trial of Botulinum Toxin Type A in Hereditary Spastic Paraplegia—The SPASTOX Trial

**DOI:** 10.1002/mds.28659

**Published:** 2021-07-24

**Authors:** Jorik Nonnekes, Alexander Geurts

**Affiliations:** ^1^ Donders Institute for Brain, Cognition and Behaviour, Department of Rehabilitation Radboud University Medical Centre, Center of Expertise for Parkinson & Movement Disorders Nijmegen the Netherlands

In a recent issue of *Movement Disorders*, Diniz de Lima and colleagues^1^ report the results of a double‐blind randomized placebo‐controlled crossover trial on the effects of botulinum toxin type A (BoNT‐A) injections—targeting the triceps surae and adductor magnus, bilaterally—in patients with hereditary spastic paraplegia (HSP). This is the first randomized controlled trial evaluating the effect of BoNT‐A in HSP. HSP is a rare condition, and we applaud the authors for successfully completing this trial and for contributing to the scientific knowledge in this field. However, we are concerned about the validity of the findings, and these concerns are related to the very essence of spasticity management.

The authors included 55 patients with various forms of HSP, including 25% with complex (and probably very diverse) phenotypes. More importantly, besides having a diagnosis of HSP, being an adult, and being able to walk 14 m, there were no inclusion criteria. This implies that patients were not selected based on a history or physical examination showing that they were actually bothered or functionally hindered by calf muscle or hip adductor spasticity during walking (eg, abrupt knee hyperextension in the stance phase or gait scissoring).^2^ Such a treatment approach is not based on internationally accepted standards.^3^ Preferably even, spasmolytic treatment of the legs should be based on some form of gait analysis to identify proper muscular targets, although we understand that this may not be possible in each center in the word. Several patients may have had forms of spasticity and gait patterns that did not require spasmolytic treatment at all. Moreover, it is generally accepted that focal spasmolytic treatment should be based on (individual) goal setting, of which there is no mention. This “global” way of selecting participants may be a very important reason for the negative results, because improper selection may have actually worsened functional performance in some participants.

Second, the authors already acknowledged that their BoNT‐A dosing was relatively low, particularly for triceps surae. However, treating only adductor magnus in the case of hip adductor spasticity is also not common practice, because specifically gracilis and adductor longus muscles may still have a significant functional impact.^4^ Moreover, injections were placed merely on the basis of palpation and anatomical landmarks. Even in experienced hands, this technique is not sufficiently reliable (more than 50% inaccuracy has been reported),^5^ in particular to target deeper muscles such as adductor magnus.

Given these concerns, also acknowledged by the authors, future controlled studies are warranted incorporating a personalized treatment approach to further evaluate the functional effects of BoNT‐A treatment in patients with HSP.

## Author Roles

Both J.N. and A.G. were involved in writing and editing of the manuscript.
